# Antiseptic Materials on the Base of Polymer Interpenetrating Networks Microgels and Benzalkonium Chloride

**DOI:** 10.3390/ijms23084394

**Published:** 2022-04-15

**Authors:** Elena Yu. Kozhunova, Galina A. Komarova, Oxana V. Vyshivannaya, Irina R. Nasimova, Anastasia E. Kuvarina, Vera S. Sadykova

**Affiliations:** 1 Faculty of Physics, Lomonosov Moscow State University, Lenin Hills 1, Bld. 2, 119991 Moscow, Russia; komarova@polly.phys.msu.ru (G.A.K.); vyshivannaya@polly.phys.msu.ru (O.V.V.); nasimova@polly.phys.msu.ru (I.R.N.); 2 Faculty of Chemistry, Lomonosov Moscow State University, Lenin Hills 1, Bld. 3, 119991 Moscow, Russia; 3 A.N. Nesmeyanov Institute of Organoelement Compounds, Russian Academy of Sciences, Vavilova St. 28, 119991 Moscow, Russia; 4 Presidium of Russian Academy of Sciences, Lenin Prospect 14, 119071 Moscow, Russia; 5 Gause Institute of New Antibiotics, Bolshaya Pirogovskaya St. 11, 119021 Moscow, Russia; nastena.lysenko@mail.ru (A.E.K.); sadykova_09@mail.ru (V.S.S.)

**Keywords:** polymers, microgels, stimuli-responsive polymers, PNIPAAm, macromolecular materials, antibacterial, benzalkonium chloride

## Abstract

Polymer microgels, including those based on interpenetrating networks (IPNs), are currently vastly studied, and their practical applications are a matter of thriving research. In this work, we show the perspective for the use of polyelectrolyte IPN microgels either as scavengers or carriers of antiseptic substances. Here, we report that poly-*N*-isopropylacrylamide/polyacrylic acid IPN microgels can efficiently absorb the common bactericidal and virucidal compound benzalkonium chloride. The particles can form a stable aqueous colloidal suspension or be used as building blocks for soft free-standing films. Both materials showed antiseptic efficacy on the examples of *Bacillus subtilis* and *S. aureus*, which was approximately equal to the commercial antibiotic. Such polymer biocides can be used as liquid disinfectants, stable surface coatings, or parts of biomedical devices and can enhance the versatility of the possible practical applications of polymer microgels.

## 1. Introduction

Over the past couple of decades, polymer microgels (micro- and nano-sized spherical hydrogel particles) have become popular in the polymer and colloidal sciences [1,2,3,4]. The peculiarity of microgels, among other things, lies in the ability to form stable colloidal aqueous dispersions resistant to sedimentation under normal conditions. Low cross-linking density and high solvent permeability enhance the interaction of the surrounding solution with active species bound to microgel polymer chains; the synthetic route is quite easy and reproducible [5].

Attention is also given to microgels with more complicated architecture, such as particles on the base of double interpenetrating networks (IPNs) [6,7,8,9,10]. In case the IPN structure consists of environment-sensitive polymers, such as poly(*N*-isopropylacrylamide) (PNIPAAm) and polyacrylic acid (PAA), the microgels may demonstrate double sensitivity. The size and conformational state of the subnetworks of such particles can be changed both by temperature and pH almost independently of each other. Likewise, the advantage of using IPN microgels containing anionic groups of AA as part of the second independent subnetwork is the fact that the charged groups are distributed uniformly over the microgel particle, and not over the surface, as is common for copolymer microgels [11]. This opens up additional possibilities for active substance impregnation into microgel particles and for controlling the resulting complex behavior.

At the same time, the search is ongoing for antiseptic preparations with antibacterial activity that could act as an alternative to traditionally used antibiotics due to the emergence and spreading of antibiotic-resistant microorganisms. Among the substances that have proven themselves as broad-spectrum antiseptics, which can be particularly distinguished ionic surfactants, are anionic (alkyl sulfates, alkyl sulfonates, etc.) and cationic (derivatives of quaternary ammonium salts) [12,13,14]. The interaction of surfactant molecules with cells leads to destabilization of the cytoplasmic membrane, to an increase in its permeability, and finally, to its destruction [15,16]. Nevertheless, the use of low-molecular-weight surfactants as disinfectants has many disadvantages, such as limits in application and effect duration and the need to reduce environmental contamination [17].

The formation of polymer complexes between surfactants and macromolecular compounds is recently considered as one of the possible routes for various antimicrobial formulations without the abovementioned negative effects. For example, in Ref. [18], multilayer films from hydrophobically modified poly(acrylic acid) (HMPA) and their complexes with cationic surfactants were used to prepare materials for antimicrobial surface coatings with a resistance to pathogens. In another work, the polymeric water-soluble antiseptic [19] was based on a cationic surfactant, benzalkonium chloride, and a copolymer of crotonic acid with *N*-vinylpyrrolidone was proposed [20]. It was found that the change in the electrokinetic potential of bacterial cells in the presence of a polymer nanosystem compared with monomeric surfactants occurs more sharply and at lower concentrations, which indicates the cooperativity of the process of interaction with cells. In a recent work [21], the water-insoluble polymer materials on the base of polystyrene sulfonate and benzalkonium chloride showed strong antibacterial and SARS-CoV-2 antiviral activities.

In this study, we have considered the use of polymer IPN microgels on the base of PNIPAAm-PAA as a polymeric substrate for the formation of antiseptic complexes with the common surfactant benzalkonium chloride (BAKCl), which is widely commercially used as a disinfectant compound [17]. IPN microgels might have several advantages over previously utilized linear polymers as carriers for antiseptic surfactant materials. For example, such systems can be used as agents for disinfection of drinking water due to the possibility of their relatively simple removal from the solution: their micron size allows one to separate microgel particles from the supernatant by centrifugation.

There are various examples of the use of microparticles, including single network microgels, as preparations with an antiseptic effect [22,23,24,25,26,27]. Even so, polyelectrolyte complexes of polymer IPN microgels with surfactants can be a worthy alternative. For example, since networks can change their conformation independently, it is possible to increase the access of surfactants to bacterial cells under conditions when the thermally sensitive NIPAAm network is collapsed, and the acrylic acid network is in a swollen state.

A separate goal of the study was in the development and study of the antibacterial properties of materials (films) obtained as a result of cross-linking of IPN microgels with surfactant molecules impregnated inside them. The mentioned materials were successfully obtained in our group earlier [28,29]. It was shown that depending on the temperature and pH of the NIPAAm-PAA IPN microgel dispersions during the preparation, macromaterials with different structure and swelling behavior could be formed. This may allow for the selection of the most suitable material for the manufacture of antibacterial films for use as antiseptic dressings.

## 2. Results and Discussion

### 2.1. The Interaction between IPN Microgels and Surfactant

**Figure 1:**
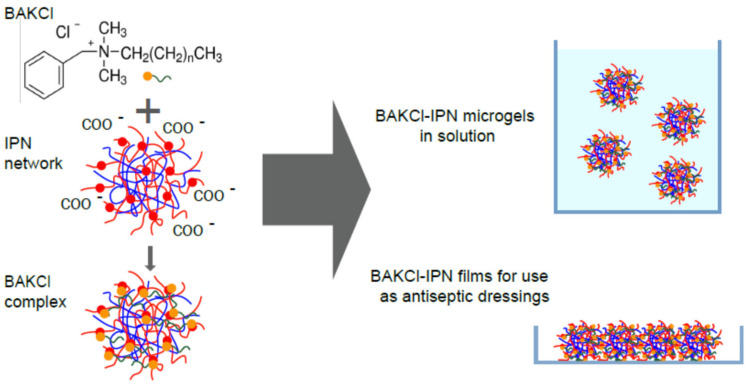
The scheme of the formation of BAKCl/IPN complexes.

For many applications, such as drug delivery or catalysis, the formation of polyelectrolyte complexes between carrier particle and an active substance is utilized. PNIPAAm-PAA IPN microgels possess negatively charged acrylic acid subnetworks as a result of protonation or deprotonation of a carboxylic group depending on medium acidity (pKa ~4.8). Accordingly, the cationic surfactant benzalkonium chloride (BAKCl), which is widely known for its antiseptic properties, serves as a guest species for the formation of the complex with IPN microgels (Figure 1). The successful appearance of such complexes was confirmed by FTIR spectroscopy (Figure S4 in Supplementary Materials).

**Figure 2:**
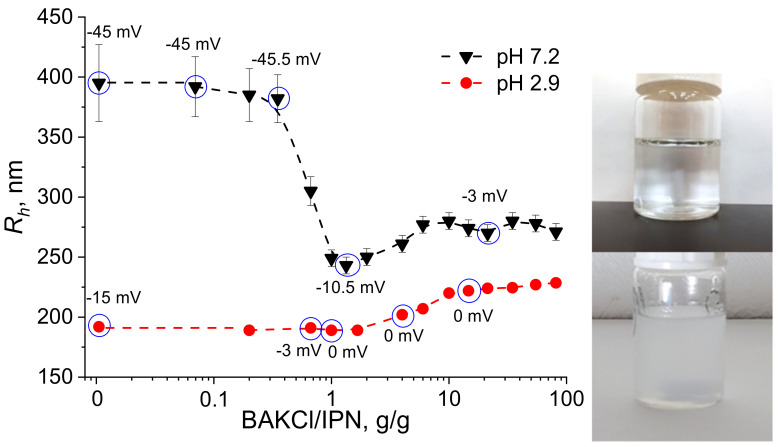
Plots of hydrodynamic radius *R_h_* versus the amount of BAKCl added to the aqueous dispersion of PNIPAAm-PAA IPN at pH 7.2 and 2.9. Zeta potential values are added above blue circles of corresponding measurements. Right image—the microgel dispersion before (upper image) and after (lower image) BAKCl addition.

The interaction between PNIPAAm-PAA IPN microgels and BAKCl surfactant was investigated—the change in particle size during absorption was monitored by the dynamic light scattering method (DLS). Typical hydrodynamic radius distributions of initial IPN microgels and BAKCl/IPN complexes are presented in Figure S1. The pH of the aqueous medium was set below and above the pKa of PAA as 2.9 and 7.2, respectively, to see the effect of the amount of charged dissociated carboxylic groups on the microgel size. The results of DLS measurements are shown in Figure 2. As can be seen at pH 7.2 (high content of dissociated groups), the addition of BAKCl to an aqueous dispersion of IPN microgels led to the significant decrease in the microgel size: hydrodynamic radius *R_h_* changed from ~400 to ~270 nm. It was also noted by the naked eye that the almost transparent solution adopted an opaque hue but remained homogeneous without a precipitate. The observed effect was caused by the binding of a cationic surfactant to deprotonated carboxylic groups of IPN microgels. Since swelling of polyelectrolyte gels and microgels is governed by the amount of charged groups in the network [30,31,32,33,34], the coupling of microgel COO^−^ with surfactant N^+^ led to the deswelling of the microgel particle. The microgels’ size ceased to decrease at the values of added surfactant of about 1 g of BAKCl per 1 g of IPN microgels (dry polymer mass). At pH 2.9, a slight increase (20%) in the hydrodynamic radius of the BAKCl/IPN complexes was observed (Figure 2). At that pH, several COOH groups could still dissociate, allowing for BAKCl molecules to bind and to increase the hydrodynamic size of the microgel particles, which was also due to hydrophobic interactions between hydrophobic parts of IPN microgels and BAKCl tails.

The estimated effectiveness of surfactant absorption was 1 mole of BAKCl per 0.9 moles of AA using the average BAKCl molecular weight equal to 372 g/mol (see Supplementary Materials for comments about BAKCl MW, C1). The value is close to the maximum possible absorption, signifying the high efficiency of the complexation, which also implies the possible use of such microgels as scavengers for similar compounds. It is possible to separate microgel particles from the supernatant by centrifugation in case of application of the substance for water purification.

For more detailed investigation, the surface charge of the IPN microgels upon addition of BAKCl was estimated by measuring the zeta (ζ) potential (Figure 2, Table S1; the BAKCl/IPN ratio for which zeta potentials were measured are marked as circles in Figure 2). It is seen that the initial ζ-potential of the microgels depended on the pH of the medium and is negative. With the increase in the added amount of BAKCl, the ζ-potential modulus value decreased toward zero because all groups that imparted negative charge to the surface of the particle were bound to positively charged surfactant molecules. The observation additionally confirmed the successful formation of polyelectrolyte complexes.

### 2.2. IPN Microgel Macromaterials with BAKCl

PNIPAAm-PAA IPN microgels can be used as building blocks for macro-sized materials [28,29]—either films or gel pieces. Such microstructured objects have their advantages and may be applied as healing bands or tissue growth matrices, for which antiseptic properties are of importance. Therefore, PNIPAAm-PAA IPN macromaterials were also studied for the possibility to absorb the benzalkonium chloride surfactant molecules (see also Scheme in Figure 1).

**Figure 3:**
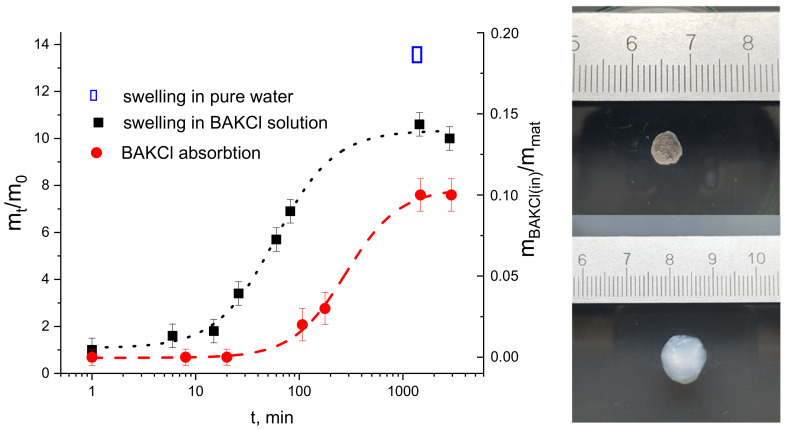
On the **left**—the kinetics of swelling (black dots and line) of the IPN material in aqueous BAKCl solution, represented as a mass of the sample *m* at the moment *t* divided by the mass of the sample at *t* = 0 (corresponds to the mass of dry material) versus time. The kinetics of BAKCl absorption (red dots and line) by IPN material, represented as a mass of the absorbed BAKCl *m_BAKCl_*_(*in*)_ divided by mass of the sample *m_mat_* versus time. On the **right**—PNIPAAm-PAA IPN material (upper image) before and (lower image) after swelling in an aqueous BAKCl solution.

First, the swelling kinetics of dry PNIPAAm-PAA IPN material in a water solution of BAKCl (*c* = 0.001 g/mL) was investigated. The pieces of dry material were placed in water solution of BAKCl and left to swell until equilibrium at room temperature. In Figure 3, the ratio of the swollen sample mass at moment *t m*(*t*) to the initial mass *m*(0) as a function of time *t* is presented. It is seen that the material rapidly swelled almost seven times and then slowly reached its equilibrium, which was 10 times to dry mass. In pure water, the swelling was bigger (almost 14 times to dry mass). The decrease in swelling of the IPN material in the BAKCl environment compared to pure water was expected and corresponds to the behavior of sole IPN microgels in similar conditions.

The effectiveness of BAKCl absorption and its kinetics was studied by the means of UV-spectroscopy (Figure 3 and Figure S2, Tables S2 and S3). For that, the sample of dry PNIPAAm-PAA IPN material (*m* = 0.0075 g) was placed in a holder and placed into 3.1 mL of aqueous BAKCl solution (*c* = 0.001 g/mL) under continuous stirring for up to two weeks, and the experiment was repeated several times. It was determined that the PNIPAAm-PAA IPN material in swollen state absorbs 0.11 ± 0.01 g of BAKCl per 1 g of dry material. Approximately, it is 0.15 mole of BAKCl per 1 mole of acrylic acid. The value is lower than that for colloidal IPN microgels due to the reduced number of available COO^−^ groups in the crosslinked material.

In our experiments, the swelling of the material took place in a water solution of cationic surfactant BAKCl. As can be seen in Figure 3 and Table S3, the PNIPAAm-PAA IPN material began to effectively absorb the surfactant from water in about 100 min, when the material swelled by about seven times. It has also been shown that the maximum uptake of cationic surfactant by the material is achieved after at least 24 h, when the state of equilibrium swelling of the material is reached.

PNIPAAm-PAA IPN microgel macromaterials can be prepared under different conditions, namely pH of the stock solution and annealing temperature. These features influence the main characteristics of the material such as swelling ratio and speed, as well as robustness because they affect the conformation of microgels directly before and during the assembly into a single piece [29]. For the presented experiments, we had chosen the films prepared from the solution with the acidity pH = 3 and drying temperature *T* = 45 °C. These parameters provide the most dense and stiff material, yet sacrifice most of the carboxylic groups due to the annealing/crosslinking process.

### 2.3. Antimicrobial Properties of IPN Microgel Materials

Bearing in mind that such composites, both in colloidal form and as a macromaterial, can be interesting as preparations in medical applications; their possible antibacterial and antifungal activities were studied.

To investigate the antimicrobial activity of colloids of BAKCl-PNIPAAm-PAA IPN microgels (BAKCl/IPN), the minimum inhibitory concentration in solutions on the series of test organisms (Table 1) was estimated. The minimum inhibitory concentration (MIC) is the lowest concentration of a chemical, usually a drug, which prevents growth of a bacteria and fungi. We determined MIC values for BAKCL-PNIPAAm-PAA IPN microgels toward collection cultures using a microtiter plate assay to understand the antibiotic potential of this material.

As it can be seen, the lowest activity was detected for *Aspergillus niger* isolate, 2 mg/L, compared to antifungal Amphotericin B with 1 mg/L. The MICs against Gram-positive bacteria ranged from 0.02 to 0.4 mg/L. In general, the antibacterial activity of BAKCl/IPN in all variants was slightly lower than that of the reference antibiotic. IPN control was not active against all test isolates. Additionally, cytotoxicity of the proposed species was investigated using MTT assay method (see comment C2 in Supplementary Materials). It was found that BAKCl/IPN composites are slightly less toxic than pure aqueous solution of BAKCl (Figure S3), meaning that the introduction of a polymeric carrier does not increase the cytotoxicity of the formulation. They could be used in antiseptic formulations when added in small amounts, since MIC is also quite low.

**Figure 4:**
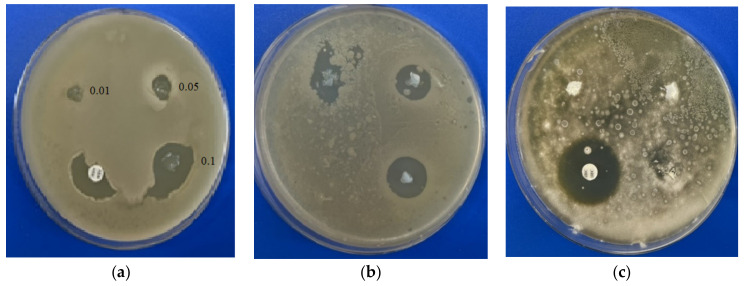
Antimicrobial activity of the films with different BAKCl concentrations (0.01–0.1) detected by disc diffusion method: (**a**) *Staphylococcus aureus* ATCC 25923; (**b**) *Bacillus subtilis* ATCC 6633; (**c**) *Aspergillus niger* ATCC 16404.

The disc diffusion test allows one to evaluate the area at which the active substance stops the bacteria/fungi from growing, which is called the inhibition zone. The comparison of the zones of inhibition allows one to compare the effectiveness of the proposed substances with commercial drugs. Using an in vitro test by the disc diffusion assay, we determined that BAKCl-PNIPAAm-PAA IPN macromaterials (BAKCl/macro) have antifungal activity against mold strains *Aspergillus niger* ATCC 16404 only in 0.1 g/g film concentration (Table 2). At the same time, these composites showed strong antibacterial effect against Gram-positive *Bacillus subtilis* and *S. aureus* in all concentrations of BAKCl. The level of growth inhibition of *Staphylococcus aureus* by BAKCl against bacteria at 0.1 g/g was similar to that of commercial antibiotic amoxiclav. Activity decreased with the decrease in BAKCl concentration in films in line from 0.1–0.01 for both bacterial tests (Figure 4), which is expected. The sizes of the inhibition zones around the pieces of BAKCl/macro in panels (a) and (b) increase with the growth of BAKCl concentration in the material, indicating the effectiveness in preventing the proliferation of the bacteria. As shown in Figure 4, the BAKCl films exhibited weaker antifungal effect than antibacterial activity.

## 3. Experimental Section

### 3.1. Materials

All materials were acquired from Sigma-Aldrich, Munich, Germany unless stated otherwise: *N*-isporopylacrylamide (NIPAAm—monomer), acrylic acid (AA—monomer), *N*,*N*′-methylenebisacrylamide (BIS—crosslinking agent), ammonium persulfate (APS—initiator), tetramethylethylenediamine (TEMED—catalyst agent), benzalkonium chloride (BAKCl—cationic surfactant). Bacterial strains *B. subtilis* ATCC 6633, *S. aureus* ATCC 25923, and the fungal strain *Aspergillus niger* ATCC 16404 were obtained from the American Type Culture Collection (ATCC, Manassas, VA, USA). Acrylic acid was purified by distillation. Other compounds were used as received. Water was purified using Millipore Milli-Q (Millipore Corp., Burlington, MA, USA) system.

### 3.2. Microgel Synthesis

PNIPAAm-PAA interpenetrating networks microgels (IPN microgels) were obtained by the formation of a PAA network inside the PNIPAAm microgels matrix (see the explanatory scheme in the Supplementary Materials, Scheme S1). For this purpose, a two-stage synthetic procedure was used.

First, PNIPAAm microgels (matrix) particles were synthesized via typical surfactant-free radical thermally initiated precipitation polymerization of NIPAAm in an aqueous solution in the presence of a crosslinking agent (BIS concentration of 1 mol.% in terms of monomer). The monomer concentration in the reaction mixture was 1 wt.%; the concentration of the initiator APS—0.07 wt.%. The polymerization took place in a nitrogen atmosphere at a temperature of 70 °C with continuous magnetic stirring at a speed of 800 rpm for 24 h. An aqueous dispersion of the synthesized microgels was gradually cooled to room temperature and purified by dialysis (pore size of dialysis bags ~18,000 kDa).

Second, for PAA network synthesis inside PNIPAAm microgels, a dispersion of PNIPAAm microgels was diluted to the concentration of 0.1 wt.% in deionized water. AA monomer (0.1 wt.%), BIS crosslinker (0.01 wt.%), and APS initiator (0.01 wt.%) were added to the dispersion. The polymerization was started by adding of the APS initiator and TEMED—catalyst agent. The polymerization of the PAA networks inside PNIPAam microgels took place in a nitrogen atmosphere at a temperature of 23 °C with continuous magnetic stirring at a speed of 800 rpm for 120 min. Aqueous dispersions of the synthesized IPN microgels were purified by dialysis from low molecular weight compounds and unreacted monomers. For the detailed mechanism of the PNIPAAm-PAA IPN structure formation, please see the following papers [7,11,35].

### 3.3. Microgel Macromaterials Preparation

The procedure for the preparation of macromaterials from IPN microgels consists of the following: the microgels dispersions were concentrated, redissolved in acidic medium, dried, and then annealed at a high temperature. Annealing is necessary for interchain crosslinking of the polyacrylic acid subnetwork of IPN microgels into a single percolating macronetwork due to the formation of the anhydrides O–RC–O–RC–O as a result of degradation of carboxyl groups. To concentrate IPN microgels, 4.7 mL of the stock solution of PNIPAAm-PAA IPN microgels was placed in a centrifuge tube and underwent centrifugation at 14,000 rpm for 10 min to separate microgel particles. The supernatant was removed from the tube, such that enough volume of water was retained to cover the sediment. Then, the sediment was dissolved using a magnetic stir bar. A homogeneous concentrated solution of microgel was obtained in 24 h. For each tube, 380 ± 20 µL of concentrated microgel solution was received from 6 mL of stock solution. After that, 6 µL of 1 M solution of H_2_SO_4_ was added. The solution was mixed with a spatula and placed in an air thermostat at 45 °C. In a typical experiment, the process of crosslinking of the sample started in one hour of heating, simultaneously with the release of water from the material. The released water was removed with a pipette. The material was left in the thermostat at 45 °C until complete drying. Then, the dry material was placed in the thermostat at 93 °C for 5 h. As a result, the PNIPAAm-PAA IPN macro-sized material (film) weighing about 0.007 ± 0.0005 g was obtained [29].

### 3.4. Introduction of BAKCl

Cationic surfactant BAKCl was introduced both into (1) single PNIPAAm-PAA IPN microgel particles in solution and (2) macromaterials prepared by microgels crosslinking.

(1) BAKCl species was added to the 0.2 wt.% aqueous solutions of PNIPAAm-PAA IPN microgels to acquire the surfactant concentration of 0.01 g/mL. The mixtures were left for 48 h, and then the microgel dispersion was purified from the unabsorbed BAKCl by several cycles of dialysis against water (dialysis bag pore size ~20,000 kDa). For antibacterial activity analysis, different concentrations of BAKCl-IPN aqueous solutions were prepared by diluting stock solutions.
(2) The samples of PNIPAAm-PAA IPN material were placed in an aqueous solution of BAKCl, where they were kept for 7 days at room temperature. Then, the samples were washed several times in pure water to remove the excess BAKCl substance. To study the antibacterial properties, films with different ratios of absorbed surfactant to the mass of the substance were prepared. For this, the samples of material about the same weight were placed in water solutions with a different concentration of BAKCl.

### 3.5. Dynamic Light Scattering

Dynamic light scattering (DLS) measurements were performed using PhotoCor Complex spectrometer (PhotoCor Instruments, Moscow, Russia) equipped with a He–Ne laser (λ = 633 nm, 10 mW) as the light source and a pseudo-cross-correlation system of photon counting. The real-time correlator was employed in a logarithmic configuration. Distributions over decay time τ and hydrodynamic radius *R_h_* were obtained using a nonlinear regularized inverse Laplace transformation method (CONTIN). The IPN/BAKCl complexes were prepared by mixing IPN aqueous dispersion (0.075 wt.%) and concentrated surfactant aqueous solution (1 and 10 wt.%) at the appropriate ratio under stirring. The samples were held for at least 30 min before measurements. Measurements of the IPN/BAKCl complexes’ hydrodynamic radii *R_h_* were carried out at a scattering angle of 90° and temperature 23 °C. All values of *R_h_* were obtained by taking the average of at least 3 measurements.

### 3.6. Zeta Potential Measurements

Zeta potential was measured using an analyzer PhotoCor Compact-Z (PhotoCor Instruments, Russia) equipped with an AlGaInP diode laser (λ = 638 nm, 30 mW) as the light source and a laser Doppler anemometer. Doppler signal analysis was performed in a mode of phase analysis light scattering (PALS). Electrophoretic mobility μ_E_ of particles was converted to zeta potential ζ using the Smoluchowski equation: μ_E_ = 2εζ/3η, where ε is the dielectric constant, and η is the solvent viscosity. All values of ζ were obtained by taking the average of at least 6 measurements at a scattering angle of 20°. The IPN/BAKCl preparation method and the concentrations were the same as in the dynamic light scattering method. The measurements were performed at 23 °C, and the samples were held for 30 min before the measurements.

### 3.7. UV-Spectrophotometry

The ability of PNIPAAm-PAA IPN material to absorb the cationic surfactant BAKCl was investigated by UV-spectrophotometry. The absorbance of BAKCl by IPN material at 263 nm was measured by UV-spectrometry using a SF-2000 spectrophotometer (“OKB-Spectr”, St. Petersburg, Russia). The sample of the material was placed in the water solution of BAKCl for several days at room temperature. After that, the absorption spectrum of the solution was obtained using the UV-spectrophotometer. The concentration of BAKCl in the solution *c* was calculated using the Beer–Lambert law:

(1) $$c=\frac{D}{\varepsilon \cdot l}$$

where *ε* is the molar extinction coefficient of BAKCl, *D* is the optical density of the solution, and *l* = 1 cm—the thickness of the quartz cuvette. The estimated extinction coefficient of BAKCl *ε* = 1170 mL cm^−1^ g^−1^ at 263 nm.

The absorbance kinetics of BAKCl by PNIPAAm-PAA IPN material was also studied. The dry material was placed in a water solution of BAKCl at 23 °C. During the experiment, the solution was mixed with a magnetic stirrer at 100 rpm. To avoid the destruction of the material during the mixing process, the material was fixed using a special holder. The holder supported the material, and it was permeable for water and BAKCl. At certain time intervals, the optical density of the solution *D* at 263 nm was measured. The BAKCl concentration at moment *t* was calculated using Equation (1).

The amount of absorbed BAKCl was calculated as the difference between concentration of species in the solution before and after the experiment (or at the moment *t* in case of kinetic measurements).

### 3.8. Antimicrobial Activity Testing

The minimum inhibitory concentrations (MIC) for Gram-positive and Gram-negative bacteria of BAKCl-enriched microgel dispersions were determined by two-fold serial microdilution method in a cation-adjusted Müller–Hinton medium for bacteria and in a liquid culture medium RPMI 1640 with L-glutamine without sodium bicarbonate for fungi in accordance with the requirements of the Institute of Clinical and Laboratory Standards (CLSI/NCCLS) for fungi [36,37,38]. The antibacterial antibiotic amoxiclav and the antifungal antibiotic amphotericin B from Sigma-Aldrich were used as reference drugs.

The spectrum of antimicrobial activity of the BAKCl-enriched macromaterials was evaluated in vitro by the disc diffusion assay as indicated [39]. Disks of 6 mm of polymer films with different active species concentrations—0.002, 0.05, 0.01 g of BAKCl per 1 g of the film—were immersed for 1 min in ethanol, dried, and placed in a Petri dish. Inhibition zones were measured manually using a digital caliper. Assays were performed three times in triplicate.

## 4. Conclusions

To summarize, we studied the ability of PNIPAAm-PAA IPN microgels to form polyelectrolyte complexes with oppositely charged antiseptic substance benzalkonium chloride. Two types of objects were investigated—IPN microgel colloids and macro-sized materials developed on the base of crosslinked IPN microgels. Both of them effectively form complexes by binding benzalkonium chloride molecules and shrink upon this complex formation with BAKCl due to polyelectrolyte swelling effect leveling. Yet, the solubility in water (in the case of individual microgel particles) remains stable. The absorption capacity of microgel colloids was evaluated to be as high as 0.9 moles of BAKCl per 1 mole of acrylic acid monomer units, also meaning that such particles could be used for the extraction of the substance from the surrounding solution.

For the first time, we showed that the introduction of BAKCl into IPN microgel colloid dispersions and macro-sized microgel films allows one to obtain the materials with pronounced antibacterial properties. Both forms—liquid aqueous dispersion and soft film—show the efficacy against *Bacillus subtilis* and *S. aureus* approximately equal to commercial antibiotic amoxiclav. These findings enhance the versatility of the possible practical applications of polymer IPN microgels, for example as agents for disinfecting of drinking water or building blocks for antibacterial dressings creation. It is also known that BAKCl-containing materials have an antiviral effect on SARS-CoV-2, opening the opportunities for the use of the studied materials in disinfection.

## Figures and Tables

**Table 1:** MIC of the used antimicrobial agent (BAKCl/IPN) against opportunistic test bacteria and fungi.

Test Strains	BAKCl/IPN mg/L	IPN Control	Amoxiclav/Clavulonic Acid 20/10 mg/L	Amphotericin B 40 mg/L
*Staphylococcus aureus* ATCC 25923	0.4	-	0.25	*
*Bacillus subtilis* ATCC 6633	0.02	-	0.01	*
*Aspergillus niger* ATCC 16404	2	-	*	1

* not tested; - no antibiotic effect.

**Table 2:** The antimicrobial activity of the films with BAKCl was measured by disc diffusion assay.

Compounds	Zone of Inhibition, mm, g BAKCl/g Film					
Test Strains	0.1	0.05	0.01	0.002	Amoxiclav/Clavulonic Acid 20/10 µg	Amphotericin B 40 µg
*Staphylococcus aureus* ATCC 25923	18	14	10	7	15	-
*Bacillus subtilis* ATCC 6633	25	15	13	11	>35	-
*Aspergillus niger* ATCC 16404	20	0	0	0	*	14

* not tested; - no antibiotic effect.

## Data Availability

The authors confirm that the data supporting the findings of this study are available within the article and its supporting materials.

## Supplementary Materials

The following supporting information can be downloaded at: https://www.mdpi.com/article/10.3390/ijms23084394/s1. References [40,41,42,43] are cited in the supplementary materials.
