# Supplementary material for: Antiseptic Materials on the Base of Polymer Interpenetrating Networks Microgels and Benzalkonium Chloride
Source: Int J Mol Sci. 2022 Apr 15;23(8):4394. doi: 10.3390/ijms23084394 (PMC9027481; doi:10.3390/ijms23084394)
Supplement: Supplementary file 1 [file ijms-23-04394-s001.zip › ijms-1663925-supplementary.pdf]

Supplementary Materials

Antiseptic materials on the base of polymer interpenetrating networks microgels and benzalkonium chloride

Elena Yu. Kozhunova[1,2*], *Galina A. Komarova*[1], *Oxana V. Vyshivannaya*[3,1], *Irina R. Nasimova*[4,1], *Anastasiya E. Kuvarina*[5], *Vera S. Sadykova*[5]

1 Faculty of Physics, Lomonosov Moscow State University, Lenin Hills, 1, bld. 2, 119991 Moscow, Russia

2 Faculty of Chemistry, Lomonosov Moscow State University, Lenin Hills, 1, bld. 3, 119991 Moscow, Russia

3 A.N. Nesmeyanov Institute of Organoelement Compounds of Russian Academy of Sciences, Vavilova St., 28, 119991 Moscow, Russia

4 Presidium of Russian Academy of Science, Lenin Prospect, 14, 119071 Moscow, Russia

5 Gause Institute of New Antibiotics, ul. Bolshaya Pirogovskaya, 11, 119021 Moscow, Russia Moscow, Russia

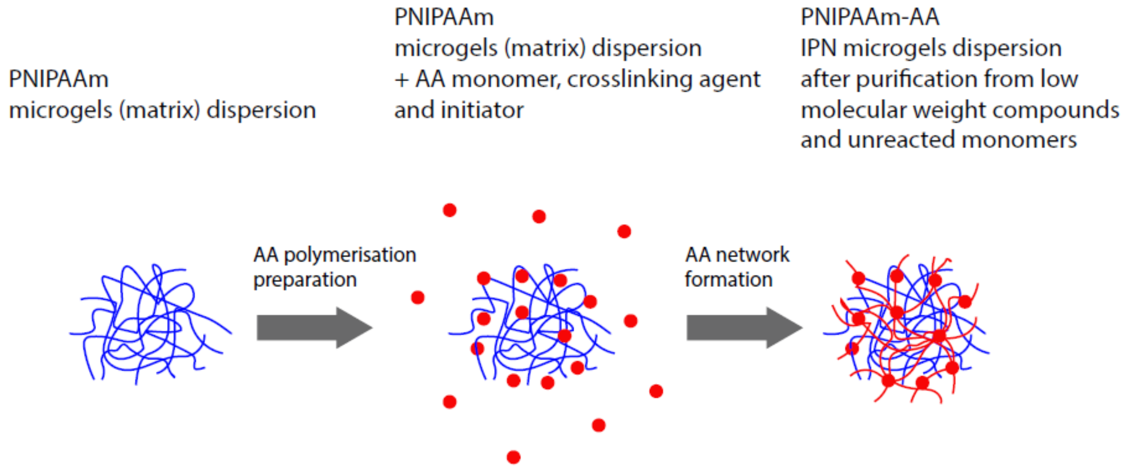

Scheme S1. The scheme of the IPN network microgel synthesis.

The basic principles of the formation of microgels by the precipitation polymerization method are described in Ref. [40]. The principles of the formation of the IPN structure in the microgels can be found in Refs. [6], [7], [9] in the main text.

C1. Regarding BAKCl molecular weight: Benzalkonium chlorides (BAKCls), also known as alkyl dimethyl benzyl ammoniumchlorides, alkyl dimethyl (phenylmethyl) quaternary ammonium chlorides, ammonium alkyl dimethyl (phenylmethyl) chlorides, or ammonium alkyl dimethyl benzyl chlorides, are a class of quaternary ammonium compounds (QACs). They are prepared and commercialized as a mixture of compounds with different lengths for the alkyl chain, ranging from C8 to C18 [17].

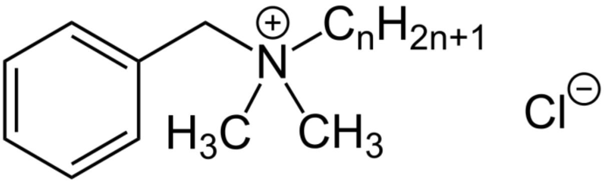

n = 8, 10, 12, 14, 16, 18

Linear formula - $C_6H_5CH_2N(CH_3)2RCl$ ($R=C_8H_{17}$ to $C_{18}H_{37}$) [41].
Average molecular weight for this manuscript was taken as equal to 372 g/mol [42].

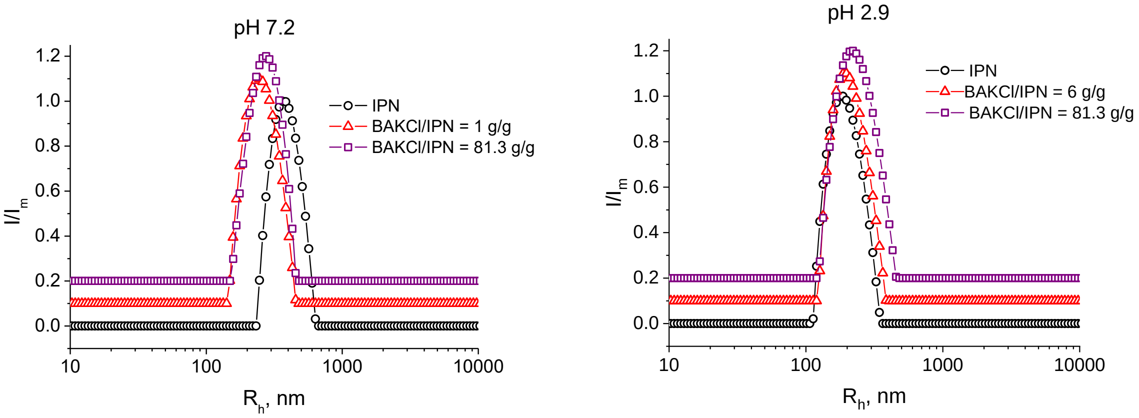

Figure S1. Hydrodynamic radius distributions for initial PNIPAAm-PAA IPN dispersions and BAKCl/IPN complexes at pH 7.2 and 2.9. The scattering angle is 90°.

Table S1. The zeta potential of IPN/BAKCl complexes at different pH.

BAKCl/IPN, g/g	Z-potential, mV	BAKCl/IPN, g/g	Z-potential, mV
	IPN-3 pH 7.2		IPN-3 pH 2.9
0	–44.9 ± 1.7	0	–15.2 ± 1.3
0.07	–45.0 ± 1.0	0.67	–3.1 ± 0.9
0.35	–45.6 ± 2.5	1	–0.7 ± 1.0*
1.33	–10.4 ± 0.1	4	–0.4 ± 1.0*
21.33	–3.1 ± 0.4	14.67	–0.1 ± 1.0*

* The measurement accuracy near 0 mV is quite low. Therefore, these three values can be considered as zero.

Table S2. Absorption of BAKCl substance by PNIPAAm-PAA IPN material in a water solution.

Sample	*days*	*m*, g	*V*, ml	*D*	c_{day}, g/ml	m_{BAKCL}(out), g	m_{BAKCL}(in), g	m_{BAKCL}(in)/*m*
1	3	0.0075	2.23	0.80	0.00068	0.,0015	0.0008	0.10
2	3	0.0075	2.77	0.84	0.00072	0.0020	0.0008	0.11
3	14	0.014	5.04	0.84	0.00072	0.0036	0.0016	0.11

m—mass of dry PNIPAAm-PAA IPN material;
V—volume of BAKCl solution;
D—optical density of solution;

c_{day}—BAKCl concentration in the solution in 3 or 14 days;
m_{BAKCl}(out)—mass of BAKCL in solution;
m_{BAKCl}(in)—mass of BAKCl in PNIPAAm-PAA IPN material;
c_0 = 0.001 g/ml is the initial concentration of BAKCl in solution.

Table S3. Absorption kinetics of BAKCl by PNIPAAm–PAA IPN material in a water solution.

t, min	*D*	c_t, g/ml	m_{BAKCL}(out), g	m_{BAKCl}(in), g	m_{BAKCl}(in)/m
0	1.27	0.0011	0.0034	0	0
8	1.27	0.0011	0.0034	0	0
20	1.27	0.0011	0.0034	0	0
107	1.21	0.0010	0.0032	0.0002	0.02
177	1.19	0.0010	0.0032	0.0002	0.03
1476	0.99	0.0008	0.0026	0.0008	0.10
2931	0.99	0.0008	0.0026	0.0008	0.10

D—optical density of solution;
c_t—BAKCl concentration in solution at moment of time *t*;
m_{BAKCl}(out)—mass of BAKCl in solution;
m_{BAKCl}(in)—mass of BAKCl in PNIPAAm–PAA IPN material;
m = 0.0075 g is the mass of dry PNIPAAm–PAA IPN material.

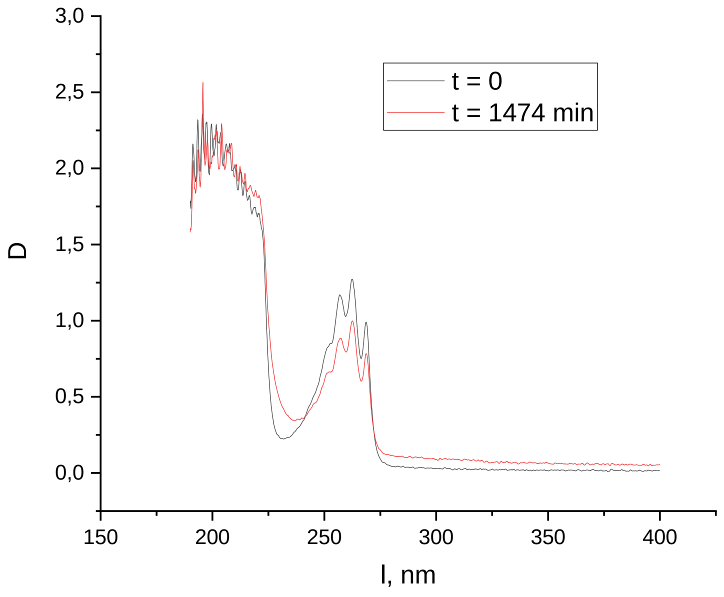

Figure S2. UV–VIS spectra of the outer BAKCl solution before and after IPN material incubation.

C2. MTT assay method

Cytotoxicity of the microgels was investigated by MTT assay [43]. Mouse pancreatic carcinoma cell lines Pan02, mouse fibroblasts L929, and human lung carcinoma A549 (collection of IBCh RAS) were grown in RPMI-1640 medium with the addition of 10% fetal calf serum (FCS, GE Healthcare, Chicago, IL, USA), penicillin, streptamycin, and glutamine (PanEco, Moscow, Russian Federation). Cells were removed from the plates with a solution of 0.05% trypsin–EDTA (PanEco, Moscow), and their concentration was determined. The gels and benzalkonium chloride

sample were diluted to yield a 10-2430 dilution. The cells were seeded at 10,000 cell/well of flat-bottom 96-well plates (Costar, Washington, WA, USA) and incubated for 72 h in a CO_2-incubator at 37 °C. MTT (Sigma, Merck KGaA, Darmstadt, Germany) was added to each well for the last 4 h. Culture medium was discarded from the wells, and formazan crystals were dissolved in 100 μL of DMSO for 20 minutes. The optical density was read on the spectrophotometer (ThermoScientific, Waltham, MA, USA) at 540 nm. The results were analyzed by the Excel package (Microsoft). Cytotoxic concentration giving 50% of the maximal toxic effect (IC_{50}) was calculated from the titration curves. The inhibition of proliferation (inhibition index, II) was calculated as $II=(1 - OD_{experiment})/OD_{control}$, where OD is MTT optical density. The results are presented in **Figure S3.**

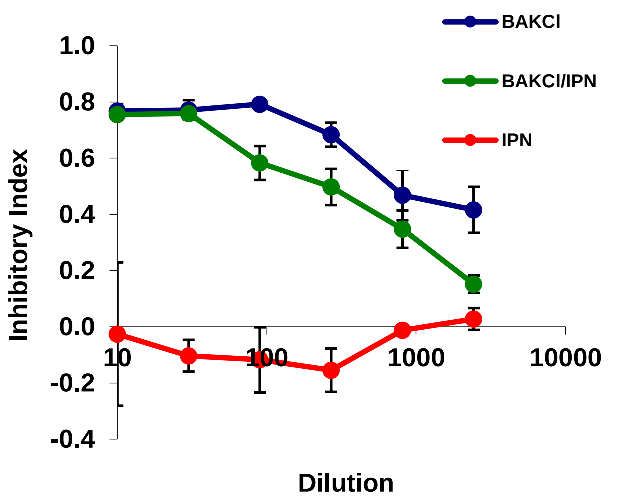

(a)

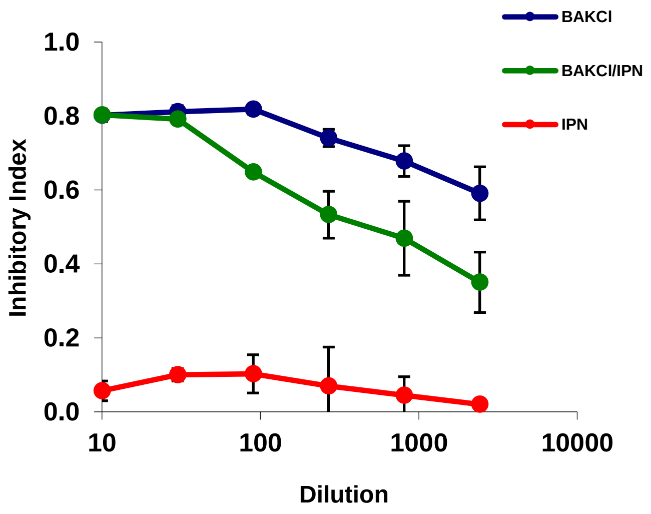

(b)

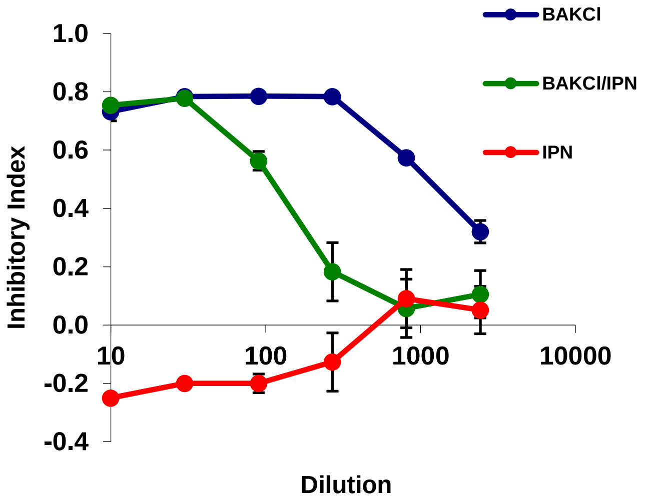

(c)

Figure S3. Cytotoxixity evaluation graphs measured by MTT assay on (a) mouse pancreatic carcinoma cell lines Pan02, (b) mouse fibroblasts L929, and (c) human lung carcinoma A549. Red line corresponds to pure IPN microgel water dispersions, blue line to benzalkonium chloride (BAKCl) aqueous solution, and green line to BAKCl/IPN material. The initial concentration of the active species was 0.1 g/l, which is much higher than MIC of the BAKCl/IPN.

C3. *FTIR measurements*

Fourier-transform Infrared spectra (FTIR) were measured on a Bruker VERTEX 70 spectrometer in the wavelength range 4000–500 cm^{-1}. The dried samples were preliminarily ground into powder in an agate mortar and pressed into KBr tablets weighing 0.2 g. The weight of the test substance in the tablet was 0.002 g.

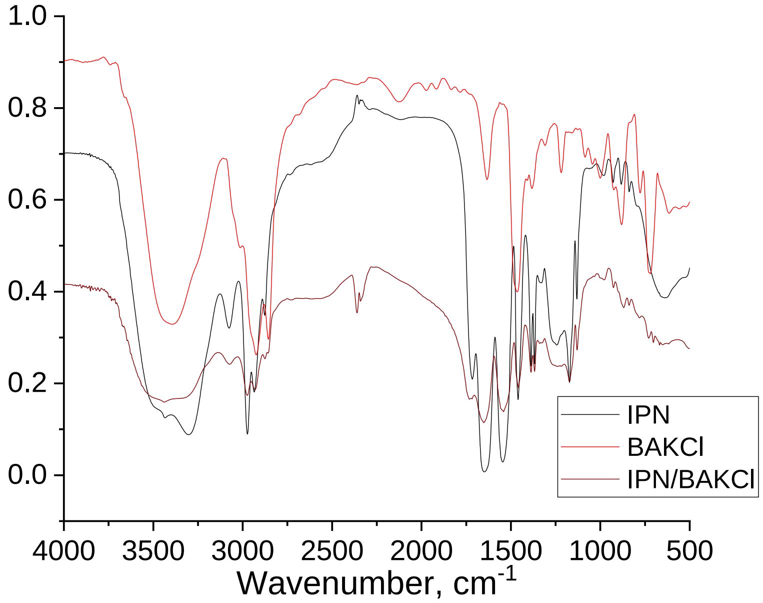

Figure S4. FTIR spectra of IPN microgels, BAKCl and IPN/BAKCl composites.
